# Maternal Glomerular Filtration Rate in Pregnancy and Fetal Size

**DOI:** 10.1371/journal.pone.0101897

**Published:** 2014-07-08

**Authors:** Nils-Halvdan Morken, Gregory S. Travlos, Ralph E. Wilson, Merete Eggesbø, Matthew P. Longnecker

**Affiliations:** 1 Department of Global Public Health and Primary Care, University of Bergen, Bergen, Norway; 2 Department of Clinical Sciences, University of Bergen, Bergen, Norway; 3 Department of Obstetrics and Gynecology, Haukeland University Hospital, Bergen, Norway; 4 Cellular and Molecular Pathology Branch, National Institute of Environmental Health Sciences, National Institutes of Health, Department of Health and Human Services, Research Triangle Park, North Carolina, United States of America; 5 Division of Epidemiology, Norwegian Institute of Public Health, Oslo, Norway; 6 Epidemiology Branch, National Institute of Environmental Health Sciences, National Institutes of Health, Department of Health and Human Services, Research Triangle Park, North Carolina, United States of America; Hôpital Robert Debré, France

## Abstract

**Background:**

The relationship of maternal glomerular filtration rate (GFR) in pregnancy to fetal size needs to be better characterized as it impacts an ongoing debate about confounding effect of maternal GFR in investigations of important environmental contaminants. We aimed to characterize the size of the association between maternal GFR and infant birth weight.

**Materials and Methods:**

A sub-cohort of 953 selected women (470 women with and 483 women without preeclampsia) in the Norwegian Mother and Child Cohort (MoBa), recruited during 2003–2007 were analyzed. GFR in the second trimester was estimated based on plasma creatinine. Birth weight was ascertained from the Medical Birth Registry of Norway. Multivariate linear regression was used to evaluate the association between maternal GFR in second trimester (estimated by the Cockroft-Gault [GFR-CG] and the modification of diet in renal disease [GFR-MDRD] formulas) and infant birth weight. Partial correlation coefficients were also calculated.

**Results:**

Maternal GFR-CG (β: 0.73 g/ml/min, p = 0.04) and GFR-MDRD (β: 0.83 g/ml/min, p = 0.04) were associated with infant birth weight in models adjusted for maternal weight in kilograms, preeclampsia, and gestational age at delivery (days). Partial correlation coefficients for the association between infant birth weight and GFR were 0.07 for both formulas. Although the birth weight-GFR association was stronger among the women with preeclampsia, the difference from women without preeclampsia was not statistically significant.

**Conclusion:**

These data support an association between GFR during pregnancy and infant birth weight, and indicate that GFR may confound selected epidemiologic associations.

## Introduction

The relationship of glomerular filtration rate (GFR) in pregnancy to fetal size needs to be better characterized because the size of the association impacts an important ongoing debate in environmental health. Perfluoroalkyl substances (PFAS) are environmental contaminants detectable in the serum of nearly everyone [1], and the relation of exposure to health outcomes is under active investigation [2], [3]. That birth weight is negatively associated with serum concentration of PFAS has been shown with remarkable consistency [4]–[7]. Because PFAS are excreted in proportion to GFR, if, as indicated in several studies, GFR is proportional to fetal size, the PFAS-birth weight association could be due to confounding by GFR rather than a toxic effect of PFAS [8]. The extent to which the PFAS-birth weight association is confounded by GFR depends on the strength of the GFR-fetal size relationship, but almost nothing is known about it.

Direct measures of GFR in pregnancy and birth weight were made in two small studies [9], [10]; these can be used to estimate the size of the GFR-birth weight relation, and give widely differing values (see below). Indirect measures of GFR in pregnancy, such as serum creatinine and uric acid, also support a positive relationship with birth weight [11]–[13]. Of the existing studies relevant to the GFR-fetal size relationship, none have quantitated it in straightforward physiologic terms such as grams per fetal weight per ml/min GFR.

In line with previous findings in the field, we hypothesized that there is an association between maternal estimated GFR (eGFR) in mid-pregnancy and fetal birth weight. The specific aim of this study was to characterize the association between maternal eGFR and fetal size at birth in a large sample of pregnant women from Norway. GFR can be measured either directly by clearance of inulin or iohexol, or estimated indirectly by using the patient’s plasma creatinine value. In this study GFR was estimated using three widely used indirect methods; the Cockroft and Gault (CG), the Modification of Diet in Renal Disease (MDRD), and the Chronic Kidney Disease Epidemiology Collaboration (CKD-EPI) [14]–[16].

## Materials and Methods

### Material

The analyzed data were obtained from a sub-study within the Norwegian Mother and Child Cohort Study (MoBa) conducted by the Norwegian Institute of Public Health [17], [18]. The majority of pregnant women giving birth in Norway during the 1999–2008 were invited to participate in MoBa at their routine ultrasound scan in gestational week 17–18 and 38.5% of invited women consented to participate. The cohort includes 108 000 children, 90 700 mothers, and 71 500 fathers. Blood samples were obtained from both parents at inclusion (in week 17–18) and from mothers and children (umbilical cord) at birth [19]. The fourth version of the quality-assured data files provided all data that were used for the present study.

The subjects in the current study were originally selected for a case-cohort study on PFAS and preeclampsia, and were enrolled in the MoBa cohort from 2003 to 2007, as described in detail elsewhere [20]. The eligibility criteria required singleton pregnancies in women with no previous live or stillbirths and a mid-pregnancy plasma sample. The present study included 470 preeclamptic patients and 483 non-preeclamptic women. The latter group of women was randomly selected from all MoBa women who met the eligibility criteria. To be included in the present analysis, women had to have complete data on gestational age at birth, birtweight, and maternal weight; 114 subjects were excluded on that basis (74 of those for missing maternal weight), leaving 953.

### Ethics Statement

The study was approved by The Ethics Committee of the Southern Healthcare Region of Norway (REK-SØR), the Norwegian Data Inspectorate and the National Institute of Environmental Health Sciences Institutional Review Board. Participants provided their written informed consent to participate in this study upon recruitment.

### Lab analyses of plasma creatinine and GFR estimation

Maternal non-fasting blood samples were collected at enrollment in the MoBa cohort (at the time of the second trimester ultrasound scan). Samples were shipped to the biobank facilities in Oslo from hospitals and maternity units throughout Norway and the majority were received and processed the day after collection [19]. Plasma was separated, aliquoted and stored in Oslo at −80 degrees Celcius until shipped to analyzing laboratories [19].

Samples were shipped on dry ice to the National Institute of Environmental Health Sciences laboratory in Durham, USA and plasma creatinine (modified Jaffé) was analyzed with an Olympus AU400e Clinical Chemistry Analyzer (Olympus America, Inc., Irvin, TX, USA), using reagents from Beckman Coulter Inc. (Irving, TX, USA). The between-assay coefficient of variation was 3%, based on 25 QA/QC specimens that were analyzed blindly (1 QA/QC specimen per batch). The mean level was 80.4 umol/l.

Plasma creatinine was used to estimate GFR based on the formula of Cockroft-Gault (CG: GFR = (140–age)×weight (kg)×1,04/serum creatinine (µmol/l)), the modification of diet in renal disease (MDRD: GFR = 175 (serum creatinine [µmol/l]×0.011312)^−1.154^×age^−0.205^×0.742) and the Chronic Kidney Disease Epidemiology Collaboration (CKD-EPI: for females with serum creatinine ≤62 µmol/L: GFR = 144 (serum creatinine [µmol/l]×0.0113/0.7)^−0.329^×(0.993)^age in years^, and for females with serum creatinine >62 µmol/L: GFR = 144 (serum creatinine [µmol/l]×0.0113/0.7)^−1.209^×(0.993)^age in years^). The latter is a recently developed equation to calculate eGFR.

### Other variables

Infant birth weight, maternal age in years at delivery, and gestational age in days was obtained from the Medical Birth Registry of Norway (MBRN). The latter was estimated based on ultrasound performed in week 18 at inclusion into the MoBa cohort. Maternal weight in kilograms was based on self-report from questionnaire 1, filled in prior to the ultrasound assessment. Preeclampsia is reported to the MBRN as a dichotomized variable. The sub-study cases were restricted to those whose diagnosis was verified after review of antenatal medical records, as described elsewhere [20].

### Statistical analysis

Linear regression models in the total cohort were adjusted for maternal weight (in kilograms), preeclampsia (yes/no) and gestational age at delivery (days). Additional adjustment for gestational length at blood draw and maternal diabetes had no effect on the results. The birth weight-GFR relationship was statistically homogeneous across preeclampsia status (p value for interaction term >0.1), and underlying assumptions for linear regression were fulfilled. Partial correlation coefficients were also calculated with adjustment for the same covariates listed above. We also performed stratified analyses on women with and without preeclampsia, adjusting for maternal weight and gestational age at delivery. The two-sided level of significance for all analyses was 0.05. All analyses were conducted using SPSS version 20.0.

## Results

The characteristics of the 953 included women are outlined in Table 1. The women were, on average, nearly 30 years old, and delivered children with a birth weight about 200 g less than the average in Norway (fhi.no), reflecting that half the pregnancies were affected by preeclampsia. The mean value for GFR based on the CG formula was higher (161.8 ml/min) than for the MDRD formula (123.7 ml/min) or the CKD-EPI formula (121.6 ml/min). Mean gestational age at blood draw was 129.8 days (18.5 weeks) with a 95% confidence interval from 117 to 145 days. When compared to the 483 women without preeclampsia, the 470 women with preeclampsia had significantly higher mean weight, and significantly lower mean gestational age and birth weight; they also had a significantly higher proportion of small-for-gestational-age infants. Only GFR estimated by the CG formula was significantly different between women with and without preeclampsia.

**Table 1:** Characteristics of 953 pregnant women in the Norwegian Mother and Child Cohort study, Norway, 2003–2007.

	Women with preeclampsia	Women without preeclampsia		The total cohort
	n = 470	n = 483		n = 953
	Mean (SD)	Mean (SD)	p-value	Mean (SD)
Maternal age (years)	28.6 (4.5)	28.9 (4.5)	0.25	28.8 (4.5)
Maternal weight (kg) in mid-pregnancy(at the time of estimated GFR)	73.9 (14.4)	69.4 (11.0)	<0.0001	71.6 (13.0)
Gestational age at blood draw (days)	129.6 (10.1)	129.9 (11.8)	0.67	129.8 (11.0)
Gestational age at delivery (days)	269.3 (20.7)	280.3 (11.8)	<0.0001	274.9 (17.6)
Creatinine (umol/l)	53.7 (13.7)	54.8 (14.0)	0.16*	54.2 (13.9)
GFR-CG^a^ (ml/min)	169.4 (53.1)	154.4 (43.1)	<0.0001	161.8 (48.9)
GFR-MDRD^b^ (ml/min)	125.8 (36.9)	121.7 (32.9)	0.07	123.7 (34.9)
GFR-CKD-EPI^c^ (ml/min)	122.2 (16.9)	120.9 (16.6)	0.22	121.6 (16.8)
Infant birth weight (g)	3117 (780)	3521 (568)	<0.0001	3322 (710)
	n (%)	n (%)		n (%)
Small-for-gestational-age(<10^th^ percentile)	116 (24.7)	55 (11.4)	<0.0001**	171 (17.9)

Means were compared with two samples T-test if not stated otherwise.

a Glomerular filtration rate estimated by Cockroft-Gault (CG) formula.

b Glomerular filtration rate estimated by modification of diet in renal disease (MDRD) formula.

c Glomerular filtration rate estimated by Chronic Kidney Disease Epidemiology Collaboration (CKD-EPI) formula.

*Mann-Whitney U-test.

**Chi-square test.

Adjusted coefficients with standard errors (SE) from multiple linear regression analysis of the association between infant birth weight and maternal eGFR in second-trimester by the CG, MDRD and the CKD-EPI formulas are outlined in table 2. The difference in infant weight for each ml/min increase in eGFR was, for the GC-formula, 0.73 g (SE 0.36 g, p<0.05); for the MDRD-formula, 0.83 g (SE 0.41 g, p<0.05), and for the CKD-EPI-formula, 0.04 g (SE 0.82 g, N.S.). The results based on the CKD-EPI-formula were not further considered. Partial correlation coefficients for the association between infant birth weight and eGFR were 0.07 (p = 0.04) for both the CG- and MDRD formula.

**Table 2:** Adjusted coefficients with standard errors (SE) from multiple linear regression analysis of the association between infant birth weight and maternal glomerular filtration rate (GFR) in second-trimester estimated by Cockroft-Gault (CG), modification of diet in renal disease (MDRD) and Chronic Kidney Disease Epidemiology Collaboration (CKD-EPI) formulas, based on data from 470 women with preeclampsia, 483 women without preeclampsia and the total cohort of 953 pregnant women from the Norwegian Mother and Child Cohort, Norway 2003–2007.

	Women with preeclampsia	Women without preeclampsia	The total cohort
	Adjusted β^a^	Adjusted β^a^	Adjusted β^b^
	(SE)	(SE)	(SE)
GFR by CG formula	1.1* (0.49)	0.24 (0.52)	0.73* (0.36)
GFR by MDRD formula	1.3* (0.58)	0.23 (0.58)	0.83* (0.41)
GFR by CKD-EPI formula	3.0* (1.3)	1.3 (1.1)	0.04 (0.82)

*significance at the 0.05 level.

a adjusted for maternal weight (kg) and gestational age in days. The unit for β with SE in gram.

b adjusted for maternal weight (kg), preeclampsia and gestational age in days. The unit for β with SE in gram.

In women with preeclampsia (n = 470) each ml/min increase in GFR (in second trimester) the infant weight at delivery increased by 1.1 gram (SE: 0.49 gram, p<0.05) when using the CG-formula and with 1.3 gram (SE: 0.58 gram, p<0.05) when using the MDRD-formula. The same analyses for women without preeclampsia (n = 483) were not statistically significant (Table 2). However, as noted above, the results were statistically homogeneous across groups with different preeclampsia status.

In Table 3 we have summarized the available data on the relationship of birth weight with estimated maternal GFR. The data are from two previous studies [10], [12] and the present analyses. All three studies indicate a relationship, with partial correlation coefficients for the two previous studies of 0.44 [10] and 0.03 [12], respectively. The β coefficients from the three studies are statistically homogeneous, regardless of whether the CG- or MDRD-based estimate from the present study is considered. For the CG-based estimate, e.g., the χ^2^_2 d.f._ is 3.58 (p = 0.17).

**Table 3:** Relationship between glomerular filtration rate (GFR in pregnancy and birth weight, estimated in three studies.

Study	n of subjects	Gestational age (wks)when GFR estimated	mean GFR(ml/min)	β g bw/GFR_ratio_ a	SE (β)	Partial r^b^
Gibson^2^, 1973^c^	20	28	152	1603^d^	784^e^	0.44
Dunlop^4^, 1981^c^	25	26	152	67^d^	535^f^	0.03
Present study, MDRD	953	18	124	103	51	0.07
Present study, CG	953	18	162	118^g^	57	0.07

a GFR_ratio_ is the ratio of subject i’s GFR to the mean GFR. We used this metric to compare results across studies to adjust for differences in gestational week when GFR was measured.

b Partial r is partial correlation coefficient.

c measured GFR using inulin clearance.

d The beta and partial r for Gibson and Dunlop studies were calculated conditional on gestational age at birth, using the raw data in the original publications.

e Gibson had three SGA infants; their inclusion probably accounts for why that study had sufficient power to detect a birth weight-GFR relationship.

f The subjects in the Dunlop study had a narrow range of birth weights, and that may explain why the standard error is relatively large in that analysis.

g Taking the intraclass correlation coefficient (ICC) for creatinine into account gives a corrected β (SE) for the CG formula of 155 (75) (see text).

## Discussion

We found a significant association between estimated maternal GFR in the second trimester and infant birth weight by using two different formulas (CG and MDRD) for calculating eGFR in the total cohort, but not when using a third recently developed formula (CKD-EPI). The absolute values and difference in GFR between formulas were in accordance with previous studies in pregnant women [14]. When summarizing all available data on the issue, including two previous studies [10], [12] all three studies indicate a relationship, with partial correlation coefficients of 0.07, 0.44 and 0.03, for the current and the two previous studies, respectively. When stratifying on preeclampsia, we found statistically significant results only for women with preeclampsia, which confirmed that including women with preeclampsia in the total cohort increased the study power, because it increased the proportion of small-for-gestational-age (SGA) newborns in the analysis [21].

Although the estimates of the relationship between birth weight and GFR from the present two earlier studies [10], [12] appeared different, they were not only qualitatively in agreement, but they were also statistically homogeneous. Nonetheless, the coefficients for the birth weight-GFR relation in the two previous studies by Gibson [10] and Dunlop [12] seemed quite different from one another despite that inulin clearance (the gold standard method of estimating GFR) was used. Both studies were performed on limited number of patient (20 and 25, respectively). The study by Gibson [10], however, had three SGA infants and this may account for why that study had sufficient power to detect a birth weight-GFR relationship. The subjects in the Dunlop study [12] had a narrow range of birth weights, and none of the infants were SGA, and that may explain why the standard error is relatively large in that analysis. Even though eGFR is not as precise or accurate as inulin clearance, the much larger sample size and inclusion of many more SGA newborns in the present study not only strengthened the evidence that an association exists, but resulted in a direct, quantitative estimate of the size of the association, which will be of use in epidemiologic and pharmacokinetic studies. To our knowledge this is the first study to estimate the relationship between fetal size and GFR in direct, quantitative terms. While the relationship between birth weight and GFR is statistically significant, the magnitude of the relationship, as reflected intuitively by the partial correlation coefficient of 0.07, is quite modest. Nonetheless, this modest relationship may result in significant confounding in studies of birth weight in relation to PFAS concentrations.

To illustrate the importance of the birth weight-GFR relationship to epidemiologic results on birth weight and PFAS concentrations in pregnancy, we analyzed the data for the 953 subjects in a model of birth weight in relation to the concentration of perfluoroctanoic acid (PFOA), adjusted for pre-pregnancy body mass index (kg/m2), gestational age at birth, gestational weight gain, and preeclampsia. (Except for the adjustment for preeclampsia, this is the model of birth weight and PFOA in Whitworth et al. [6], fitted to data for another group of MoBa subjects. Plasma creatinine was not measured in the Whitworth et al. study; however PFAS were measured among the subjects in the present study.) Further adjustment for GFR-CG caused the PFOA coefficient to attenuate by 66% (not shown).

The estimated association between GFR and infant birth weight will tend to be influenced by random errors in measurement of plasma creatinine and more accurate assessments of the relationship would need to be based on GFR gold standard measurements rather than eGFR. Others have found an intraclass correlation coefficient of 0.76 for serum creatinine in women who had their levels measured 2 to 3 years apart [22]. Under the assumption that this translates directly into an intraclass correlation coefficient for GFR, the β after correction for this error will be 30% larger (β-CG: 0.73/0.76 = 0.96 and β-MDRD: 0.83/0.76 = 1.09) than what we observed for both the indirect formulas (Willett WC, Nutritional Epidemiology. New York: Oxford University Press. 1990, page 200). This measurement error-corrected estimate of β might be useful in pharmacokinetic models.

Pregnancy induces marked changes in renal function characterized by hyperfiltration, accompanied by systemic vasodilatation and plasma volume expansion that is fully established in mid-pregnancy [23], [24]. The GFR may increase as much as 60% compared to the pre-pregnant value [14]. Several methods are available to measure GFR, however, no consensus exists on what method is the most suitable in pregnancy [14]. The gold standard method of estimating GFR is inulin clearance, a costly and time consuming examination, unsuitable for large scale settings, like our study and in clinical settings. The most widely clinically used methods are based on single-point measurements of serum creatinine with formulas for calculation of GFR (CG and MDRD). Both the direct method (inulin clearance) and the indirect methods (CG and MDRD) underestimate GFR in pregnancy [14]. However, differences between inulin-based GFR and GFR obtained from single-point measurements of serum creatinine (CG and MDRD) stayed the same with increasing GFR [14]. It is therefore unlikely that the bias in the CG and MDRD GFR estimates will have substantially affected our estimation of the association between GFR and birth weight. The CKD-EPI formula showed no significant association with birth weight in the total cohort, but only when the women with preeclampsia were analyzed alone. This formula is an even poorer predictor of GFR in some groups [25], especially pregnant woman [26], which may explain the divergence in results.

The major strength of our data was the large sample of women who had their GFR estimated compared to the previous studies [9], [10], and the relatively large proportion of newborns who were SGA. Despite the strengths, our data have some limitations. Data on maternal weight were obtained from questionnaires and were self-reported. In general, self-reported weight is fairly accurate and precise, and that might be even more true during pregnancy [27]–[29]. Shipment of blood samples for a day at ambient temperature may have affected creatinine values in this study. The delayed separation of whole blood and ambient temperature may have increased levels of plasma creatinine [30]. Thus, our estimates of GFR may have been too low and more imprecise than usual; however, the effect on our estimate of the birth weight-GFR relationship would be to bias it towards the null. The most important weakness of the present study, however, was that the estimate of GFR used was imprecise compared with direct measures of GFR, based on clearance. All the formulas for calculation of GFR based on serum creatinine (CG, MDRD and EPI-CKD) are based on large cohorts of non-pregnant patients with at least mild renal insufficiency [26] and appear to be insufficient for estimating GFR in pregnant women [15], [31] and healthy individuals [32]. Particularly, the MDRD formula systematically underestimates GFR at higher values (above 60 ml/min/1.73 m^2^) [16]. A formal calculation of the effect of the imprecision on our β is beyond the scope of the present study, but suppose, for the sake of argument, that the true β was underestimated by about 100%. Assuming that the true β is 2 g/ml/min and the standard deviations of birth weight and GFR-CG are as given in table 1 (women without preeclampsia), with 0.80 power and a two-sided alpha of 0.05, 343 patients would need to be studied with a gold standard method (http:/Hedwig.mgh.harvard.edu/sample_size/js/js_associative_quant.html). This number could be decreased by enriching the sample with SGA infants [21], which could perhaps be done using mid-pregnancy ultrasound.

## Conclusion

These data support a modest, positive association between GFR during pregnancy and infant birth weight, and indicate that GFR may confound selected epidemiologic associations. The quantitative estimate of the relationship presented, although provisional until better estimates become available, will also inform pharmacokinetic studies of the extent of such confounding.
