# Correction: Maternal Glomerular Filtration Rate in Pregnancy and Fetal Size

**DOI:** 10.1371/journal.pone.0130752

**Published:** 2015-06-17

**Authors:** Nils-Halvdan Morken, Gregory S. Travlos, Ralph E. Wilson, Merete Eggesbø, Matthew P. Longnecker

There are errors in the last sentence of the Results section. The correct sentence is: For the CG-based estimate, e.g., the χ^2^_2 d.f._ is 3.38 (p = 0.18).

There are errors in Table 3. Please see the corrected Table 3 here.

**Table 3:** Relationship between glomerular filtration rate (GFR) in pregnancy and birth weight, estimated in three studies.

Study	n of subjects	Gestational age (wks) when GFR estimated	mean GFR (ml/min)	β g bw/GFR_ratio_ ^a^	SE (β)	Partial r^b^
Gibson^2^, 1973^c^	20	28	152	1603^d^	784^e^	0.44
Dunlop^4^, 1981^c^	25	26	152	67^d^	535^f^	0.03
Present study, MDRD	953	18	124	101	51	0.07
Present study, CG	953	18	162	125^g^	58	0.07

^a^ GFR_ratio_ is the ratio of subject i’s GFR to the mean GFR. We used this metric to compare results across studies to adjust for differences in gestational week when GFR was measured.

^b^ Partial r is partial correlation coefficient.

^c^ measured GFR using inulin clearance.

^d^The beta and partial r for Gibson and Dunlop studies were calculated conditional on gestational age at birth, using the raw data in the original publications.

^e^ Gibson had three SGA infants; their inclusion probably accounts for why that study had sufficient power to detect a birth weight-GFR relationship.

^f^ The subjects in the Dunlop study had a narrow range of birth weights, and that may explain why the standard error is relatively large in that analysis.

^g^ Taking the intraclass correlation coefficient (ICC) for creatinine into account gives a corrected β (SE) for the CG formula of 164 (77) (see text).
